# Breast Cancer Surgery at a Tertiary Center in Greece: Clinicopathological Associations and Patient Outcomes

**DOI:** 10.7759/cureus.87546

**Published:** 2025-07-08

**Authors:** Stefanos Flindris, Chrysoula Margioula-Siarkou, Georgia Margioula-Siarkou, Georgios Markozannes, Elif Empliouk, Dimitra Tasiou, Emmanouela - Aliki Almperi, Konstantina Mponiou, Stamatios Petousis, Konstantinos Dinas

**Affiliations:** 1 2nd Department of Obstetrics and Gynecology, Aristotle University, School of Medicine, Thessaloniki, GRC; 2 Department of Hygiene and Epidemiology, University of Ioannina, School of Medicine, Ioannina, GRC; 3 Radiation Oncology Unit, Theageneio Anticancer Hospital, Thessaloniki, GRC

**Keywords:** breast cancer, breast‐conserving surgery, clinical stage, mastectomy, molecular subtype, survival

## Abstract

Introduction

Breast-conserving surgery (BCS) and mastectomy are the cornerstone surgical options for invasive breast cancer, yet their comparative effectiveness in real-world practice, especially when stratified by molecular subtype, remains inadequately characterized. In light of this, we conducted a single-center retrospective cohort study to evaluate clinicopathological features, treatment patterns, and overall survival (OS) among patients undergoing lumpectomy versus mastectomy at a tertiary care center in Greece.

Methods

A total of 119 women treated between 2010 and 2020 were included: 79 (66.4%) underwent lumpectomy and 40 (33.6%) underwent mastectomy. Clinicopathological variables [tumor size, histologic grade, stage, lymphovascular invasion (LVSI), nodal status, molecular subtype] and adjuvant therapies [chemotherapy, radiotherapy, sentinel lymph node biopsy (SLNB), axillary lymphadenectomy] were compared between groups. Survival analyses were performed using Kaplan-Meier estimates with log-rank tests and a multivariable Cox proportional hazards regression.

Results

Compared with the lumpectomy group, patients selected for mastectomy more frequently presented with tumors >2 cm [29/40 (72.5%) vs. 21/79 (26.6%), p<0.001], grade 3 disease [10/40 (25.0%) vs. 21/79 (26.6%), p=0.006], stage ≥2 [31/40 (77.5%) vs. 19/79 (24.0%), p<0.001], LVSI [20/40 (50.0%) vs. 21/79 (26.6%), p=0.011], and nodal involvement [20/40 (50.0%) vs. 9/79 (11.4%), p<0.001]. The distribution of molecular subtypes differed between groups (p=0.037), with luminal B-HER2 being most common overall [46/119 (38.7%)]. Radiotherapy was administered to all lumpectomy patients [79/79 (100.0%)] versus 10/40 mastectomy patients (25.0%, p<0.001). Kaplan-Meier analysis showed superior OS in the lumpectomy cohort (log-rank p=0.001); however, multivariable adjustment identified only higher clinical stage as an independent predictor of mortality [hazard ratio (HR) per stage increment: 8.32; 95% confidence interval (CI): 2.28-30.38; p<0.01], whereas type of surgery did not remain significant.

Conclusions

In our cohort of 119 patients, BCS yielded excellent survival outcomes in early-stage, lower-risk tumors, whereas mastectomy was more often reserved for more advanced disease. Clinical stage at diagnosis, rather than surgical approach per se, emerged as the primary determinant of OS. Integration of molecular subtype into surgical decision-making may further refine personalized treatment strategies.

## Introduction

Breast cancer remains the most diagnosed malignancy and the leading cause of cancer-related mortality among women worldwide, with an estimated 2.3 million new cases and 685,000 deaths in 2020 alone [1]. Over the last four decades, advances in screening, systemic therapies, and surgical techniques have transformed long-term outcomes: what was once treated almost exclusively with radical mastectomy is now often managed with breast-conserving surgery (BCS) plus radiotherapy [2]. Seminal randomized trials, most notably NSABP B-06, demonstrated equivalent overall survival (OS) between lumpectomy plus irradiation and total mastectomy for early-stage invasive breast cancer, a finding subsequently confirmed by the Early Breast Cancer Trialists’ Collaborative Group meta-analysis [3,4]. Today, international guidelines uniformly endorse BCS as the preferred approach for patients with tumors ≤5 cm and clear margins, owing to its oncologic safety, reduced morbidity, and superior cosmetic and quality-of-life outcomes [5].

While traditional clinicopathological factors such as tumor size, histologic grade, lymph node status, and patient age continue to guide surgical decision-making, growing recognition of breast cancer heterogeneity has led to the adoption of intrinsic molecular subtypes (luminal A, luminal B HER2-, luminal B HER2+, HER2-enriched, and basal-like). These subtypes carry distinct prognostic and predictive implications. Luminal A tumors portend the most favorable outcomes; basal-like (triple-negative) cancers are associated with higher rates of local recurrence and early distant metastasis; and HER2-enriched tumors derive considerable benefit from targeted therapy [6]. However, the interaction between molecular subtype and choice of surgical procedure and their joint impact on survival remains underexplored, especially outside of large cooperative‐group settings [6,7].

Concurrently, our understanding of breast cancer has evolved from a purely histopathologic classification toward intrinsic molecular subtypes such as luminal A, luminal B (HER2-negative and HER2-positive), HER2-enriched, and basal-like, each defined by distinct gene-expression profiles and carrying prognostic and predictive implications [6,8]. Patients with luminal A tumors generally experience the most favorable outcomes, whereas those with basal-like and HER2-enriched subtypes face higher risks of early recurrence and poorer survival in the absence of targeted therapies [7]. Importantly, subtype-specific patterns of local recurrence and differential response to systemic agents suggest that intrinsic biology may interact with surgical extent to influence long-term control [7].

In routine clinical practice, the selection of lumpectomy versus mastectomy often hinges on tumor-centric features and patient preference, with less emphasis on molecular phenotype. However, subtype-specific risk profiles could inform more individualized surgical strategies; for instance, the increased propensity for locoregional failure in basal-like disease might justify a wider excision or closer postoperative surveillance, whereas luminal A tumors could be ideally suited to BCS [7,9]. Moreover, it is unclear whether the long-term survival equivalence demonstrated in trials equally applies across all intrinsic subtypes when evaluated in real-world, single-institution cohorts [9].

Despite these insights, few real-world studies have integrated molecular profiling into the assessment of surgical outcomes. It remains unclear whether the equivalence of BCS and mastectomy observed in unselected cohorts holds across all subtypes or whether certain molecular groups might benefit from extensive resection. In this single-center retrospective cohort of 119 patients with primary invasive breast cancer, we compare clinicopathological characteristics, treatment patterns, and OS between lumpectomy and mastectomy, also with stratification by molecular subtype. By combining traditional prognostic factors with contemporary molecular classification, we aim to refine patient selection for breast-conserving versus radical surgery and to address aspects related to personalized, biology-driven surgical decision-making.

## Materials and methods

Study design and patient population

We conducted a retrospective, single-center cohort study involving women diagnosed with primary invasive breast carcinoma and treated surgically between January 2018 and December 2023 at the 2nd Department of Obstetrics and Gynecology, General Hospital of Thessaloniki “Ippokrateio.” The study was conducted in accordance with the Declaration of Helsinki. Informed consent was waived, given the retrospective chart review design of the study.

Inclusion and exclusion criteria

Eligible patients were ≥18 years old with histologically confirmed invasive breast carcinoma who underwent either BCS (lumpectomy) plus whole-breast radiotherapy or mastectomy (with or without immediate reconstruction). Patients were excluded if they had bilateral or multicentric tumors precluding BCS, presented with distant metastases at diagnosis, received neoadjuvant radiotherapy, or had incomplete clinicopathological or follow-up data.

Clinicopathological data collection

Demographic and tumor data were retrieved from medical records, including age at diagnosis, menopausal status, tumor size (≤2 cm, 2-5 cm, >5 cm), histologic grade (Nottingham grading system I-III), and American Joint Committee on Cancer (AJCC) stage (0-IV) based on pathologic T and N categories. Lymphovascular invasion (LVSI) and multifocality were recorded as binary variables. Lymph node status was determined by sentinel lymph node biopsy (SLNB) and/or axillary lymphadenectomy.

Molecular subtyping

Formalin-fixed, paraffin-embedded tumor specimens were reviewed for estrogen receptor (ER), progesterone receptor (PR), and HER2 status by immunohistochemistry (IHC) and in situ hybridization (ISH) for equivocal HER2 (IHC 2+). The Ki-67 index was recorded when available. Intrinsic molecular subtypes were assigned as follows: luminal A: ER+ and/or PR+, HER2-, Ki-67 <20%, luminal B - HER2-: ER+ and/or PR+, HER2-, Ki-67 ≥20%, luminal B - HER2+: ER+ and/or PR+, HER2+, HER2-enriched: ER-, PR-, HER2+ and Basal-like: ER-, PR-, HER2-.

Treatment details

All surgical procedures, whether breast‐conserving (lumpectomy) or modified radical mastectomy, with SLNB or full axillary lymphadenectomy, were performed by our dedicated oncoplastic breast surgery team. Surgical plans and adjuvant treatment recommendations were determined at weekly multidisciplinary tumor board meetings, which include a medical oncologist, a radiation oncologist, a radiologist, and a pathologist. Adjuvant chemotherapy regimens such as anthracycline- and/or taxane-based chemotherapy, endocrine therapy (tamoxifen or aromatase inhibitors), anti-HER2 agents (trastuzumab ± pertuzumab), and radiotherapy (whole-breast or chest-wall and/or regional nodal irradiation) were prescribed according to these consensus recommendations, international guidelines and institutional protocols [5].

Follow-up and outcome measures

Patients were followed up from the date of surgery until death or last clinical contact. The primary endpoint was OS, defined as the time from surgery to death from any cause. Secondary endpoints included patterns of adjuvant treatment by surgical group and survival stratified by molecular subtype.

Statistical analysis

Analyses were performed using Stata version 16.1 (StataCorp, College Station, TX). Continuous variables are presented as median and interquartile range (IQR) and compared using the Wilcoxon rank-sum test. Categorical variables are reported as counts and percentages and compared using χ² or Fisher’s exact tests, as appropriate. Survival curves were estimated by the Kaplan-Meier method and compared using the log-rank test. A multivariable Cox proportional hazards model was constructed to evaluate the independent association of surgery type, age (per year), grade (per unit), and stage (per unit) with OS; hazard ratios (HRs) and 95% confidence intervals (CIs) were reported. Proportional hazards assumptions were verified by Schoenfeld residuals. All tests were two-sided, and a p-value <0.05 was considered statistically significant.

## Results

A total of 119 patients were included in the study: 79 (66.4%) undergoing BCS (lumpectomy) and 40 (33.6%) treated with mastectomy. Baseline clinicopathologic features and the results of statistical analysis are summarized in Table 1. The median age was slightly higher among the mastectomy group (62 years, IQR: 52-72.3) compared to the lumpectomy group (58 years, IQR: 52-69), but the difference was not statistically significant (p=0.353).

**Table 1:** Baseline clinicopathologic characteristics of patients undergoing lumpectomy versus those receiving mastectomy Continuous variables are compared using the Wilcoxon rank-sum test; categorical variables are compared using the χ² test or Fisher’s exact test (FET), so that rows with any expected cell count <5 use Fisher’s exact test (“FET” in the "Test statistic" column), while the others retain χ². The numbers in parentheses after the χ² (e.g., χ²(1), χ²(2)) are simply the degrees of freedom (df) for that particular test. Test statistics (Wilcoxon W or χ²) are shown in the “Test statistic” column IQR: interquartile range; LVSI: lymphovascular invasion

Characteristic	Lumpectomy (n=79, 66.4%)	Mastectomy (n=40, 33.6%)	Total (n=119, 100%)	Test statistic	P-value
Age, years, median (IQR)	58 (52–69)	62 (52–72.3)	61 (52–69)	W=1415	0.353
Tumor size, n (%)				FET	<0.001
≤2 cm	58 (73.4)	11 (27.5)	69 (58.0)		
2–5 cm	21 (26.6)	23 (57.5)	44 (37.0)		
>5 cm	0 (0.0)	6 (15.0)	6 (5.0)		
Grade, n (%)				χ²_(2)_ = 10.22	0.006
1	16 (20.3)	0 (0.0)	16 (13.4)		
2	42 (53.2)	30 (75.0)	72 (60.5)		
3	21 (26.6)	10 (25.0)	31 (26.1)		
Stage, n (%)				FET	0.002
0	3 (3.8)	0 (0.0)	3 (2.5)		
I	57 (72.2)	9 (22.5)	66 (55.5)		
II	17 (21.5)	17 (42.5)	34 (28.6)		
III	1 (1.3)	8 (20.0)	9 (7.6)		
IV	1 (1.3)	6 (15.0)	7 (5.9)		
Ductal carcinoma, n (%)				χ²_(1)_ = 6.20	0.013
No	15 (19.0)	1 (2.5)	16 (13.4)		
Yes	64 (81.0)	39 (97.5)	103 (86.6)		
Lobular carcinoma, n (%)				FET	0.058
No	67 (84.8)	39 (97.5)	106 (89.1)		
Yes	12 (15.2)	1 (2.5)	13 (10.9)		
Multifocality, n (%)				FET	1.000
No	74 (93.7)	38 (95.0)	112 (94.1)		
Yes	5 (6.3)	2 (5.0)	7 (5.9)		
LVSI, n (%)				χ²_(1)_ = 6.45	0.011
No	58 (73.4)	20 (50.0)	78 (65.5)		
Yes	21 (26.6)	20 (50.0)	41 (34.5)		
Neoadjuvant chemotherapy, n (%)				χ²_(1)_ = 6.10	0.013
No	67 (84.8)	26 (65.0)	93 (78.2)		
Yes	12 (15.2)	14 (35.0)	26 (21.8)		
Positive lymph nodes, n (%)				χ²_(1)_ = 21.47	< 0.001
No	70 (88.6)	20 (50.0)	90 (75.6)		
Yes	9 (11.4)	20 (50.0)	29 (24.4)		
Chemotherapy, n (%)				χ²_(1)_ = 4.02	0.045
No	45 (57.0)	15 (37.5)	60 (50.4)		
Yes	34 (43.0)	25 (62.5)	59 (49.6)		
Radiotherapy, n (%)				χ²_(1)_ = 79.22	< 0.001
No	0 (0.0)	30 (75.0)	30 (25.2)		
Yes	79 (100.0)	10 (25.0)	89 (74.8)		
Sentinel-node biopsy, n (%)				χ²_(1)_ = 36.94	< 0.001
No	2 (2.5)	19 (47.5)	21 (17.6)		
Yes	77 (97.5)	21 (52.5)	98 (82.4)		
Axillary lymphadenectomy, n (%)				χ²_(1)_ = 47.77	< 0.001
No	64 (81.0)	6 (15.0)	70 (58.8)		
Yes	15 (19.0)	34 (85.0)	49 (41.2)		
Molecular subtype, n (%)				FET	0.017
Basal-like	2 (2.5)	3 (7.5)	5 (4.2)		
HER2-enriched	12 (15.2)	6 (15.0)	18 (15.1)		
Luminal A	19 (24.1)	11 (27.5)	30 (25.2)		
Luminal B – HER2+	19 (24.1)	1 (2.5)	20 (16.8)		
Luminal B – HER2–	27 (34.2)	19 (47.5)	46 (38.7)		

Tumor characteristics

Tumor size and stage had a strong influence on surgical choice (both p<0.001). Lesions ≤2 cm were treated predominantly with lumpectomy (73.4% vs. 27.5%), whereas tumors of 2-5 cm and >5 cm were more often managed by mastectomy (57.5% and 15.0%, respectively). Likewise, early‐stage disease (stage 0-I) comprised 76.0% of lumpectomies versus 22.5% of mastectomies, while stages II-IV accounted for 77.5% of mastectomy cases versus 24.0% after lumpectomy. Histologic grade also differed (p=0.006): all grade 1 tumors were treated with lumpectomy, and grade 2-3 disease was more common in the mastectomy cohort.

Pathology and nodal status

Invasive ductal carcinoma was seen in 97.5% of mastectomy specimens versus 81.0% of lumpectomies (p=0.013), while lobular histology was more frequent after lumpectomy (15.2% vs. 2.5%; p=0.036). Lymphovascular invasion occurred in 50.0% of mastectomy patients versus 26.6% of lumpectomy patients (p=0.011), and rates of multifocality did not differ (p=0.771). Neoadjuvant chemotherapy (NAC) was administered to 35.0% of mastectomy patients compared with 15.2% of those undergoing lumpectomy (p=0.013), and pathologic node‐positivity was significantly higher in the mastectomy group (50.0% vs. 11.4%; p<0.001).

Adjuvant therapies

Chemotherapy was administered significantly more often in the mastectomy cohort (62.5% vs. 43.0%; p=0.045), whereas radiotherapy was nearly universal after lumpectomy (100.0% vs. 25.0%; p<0.001), reflecting standard guideline-driven practice. Sentinel‐node biopsy was performed in 97.5% of lumpectomy cases compared with 52.5% of mastectomies (p<0.001), while axillary lymphadenectomy was far more common following mastectomy (85.0% vs. 19.0%; p<0.001).

Molecular subtypes

The distribution of molecular subtypes differed between groups (p=0.037). Luminal B (HER2-) disease was the most frequent overall and particularly enriched in the mastectomy group (47.5%). Luminal A and luminal B (HER2+) tumors were more evenly represented, and basal-like cancers remained rare but slightly more common in mastectomy patients (7.5% vs. 2.5%).

Multivariable Cox regression analysis

The multivariable Cox regression analysis identified clinical stage as the only significant predictor of overall survival, with each increase in stage associated with a markedly higher risk of death (HR: 8.32; 95% CI: 2.28-30.38; p<0.01) (Table 2). In contrast, the type of surgery (mastectomy vs. lumpectomy), age, and tumor grade did not show statistically significant associations with survival, indicating that these factors were not independently predictive of outcomes in this cohort after adjustment for other variables (Table 2).

**Table 2:** Multivariable Cox proportional hazards model for overall survival Hazard ratios (HR) with 95% confidence intervals (CI) and p-values are shown for the surgical group, age, tumor grade, and stage

Covariate	HR	95% CI	P-value
Surgery			
Lumpectomy	Reference		
Mastectomy	2.27	0.20–25.30	0.5
Age, per year	1.02	0.92–1.12	0.7
Grade, per unit	0.5	0.08–3.31	0.47
Stage, per unit	8.32	2.28–30.38	<0.01

Survival outcome

**Figure 1:**
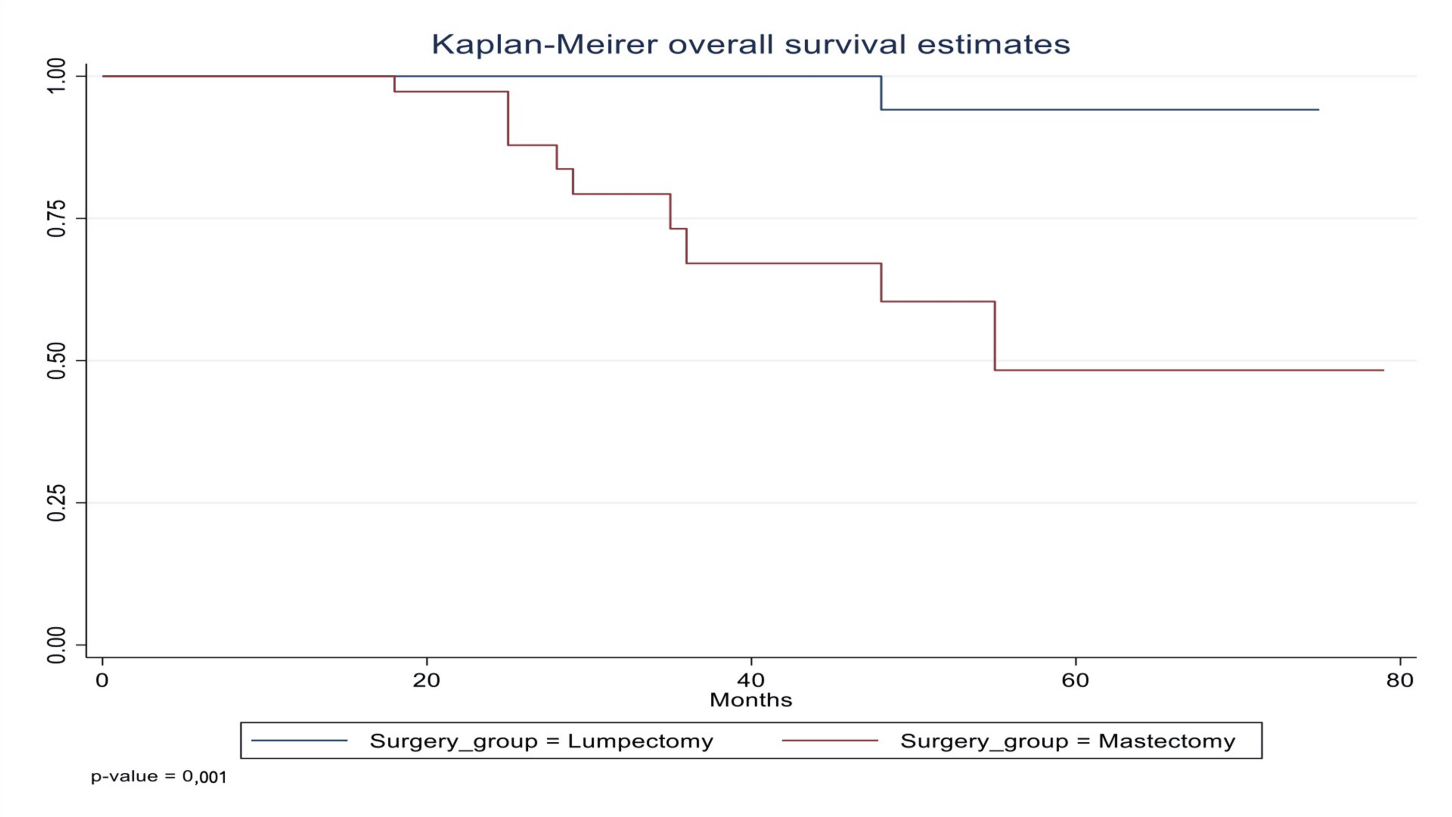
Kaplan-Meier overall survival curves for lumpectomy (blue) versus mastectomy (red) groups (p<0.001) Patients receiving lumpectomy maintain near-100% survival, while the mastectomy cohort exhibits a progressive decline over 75 months

The Kaplan-Meier analysis revealed a highly significant difference in overall survival between the two surgical groups (p<0.001). Patients treated with lumpectomy maintained near-100% survival throughout follow-up, whereas those undergoing mastectomy showed a progressive decline in survival (Figure 1). Although this separation suggests an apparent survival advantage for lumpectomy in our cohort, substantial baseline imbalances, most notably more advanced stage, higher grade, and greater nodal involvement in the mastectomy group, may drive this finding. Therefore, these unadjusted results must be interpreted with caution, and confirmatory multivariable analysis (adjusting for stage, grade, subtype, and adjuvant treatments) is required to determine whether lumpectomy independently predicts better survival.

**Figure 2:**
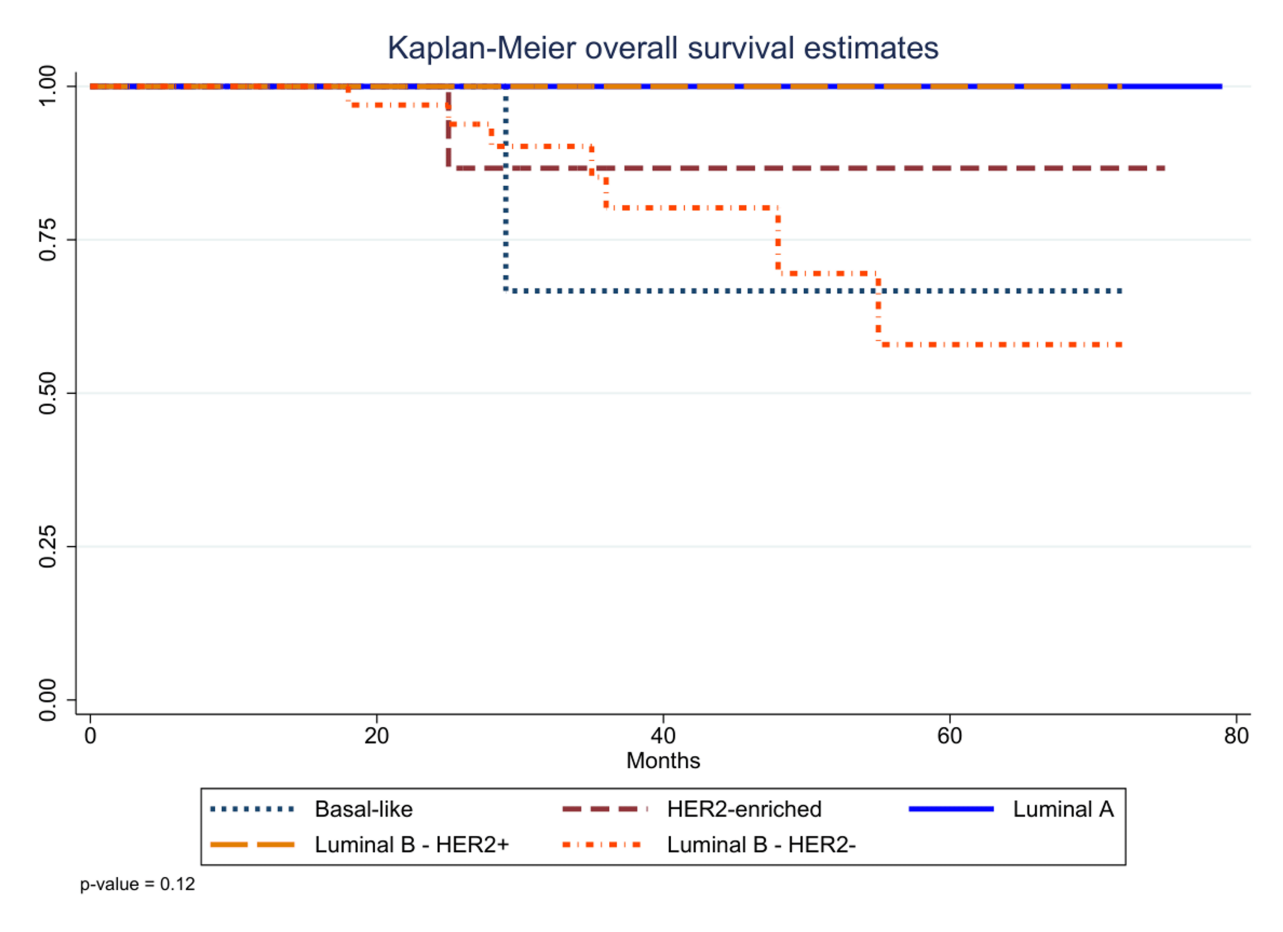
Kaplan-Meier overall survival stratified by molecular subtype (p=0.12)

As shown in Figure 2, another Kaplan-Meier estimate stratified by molecular subtype demonstrated that both luminal B-HER2+ and HER2-enriched groups experienced 100% overall survival, with no events during up to 75 months of follow-up. Luminal A and luminal B-HER2- subtypes showed modest decline, maintaining survival above approximately 80% at last follow-up. Basal-like tumors had the poorest outcomes, with survival decreasing to roughly 60% by 75 months. These subtype‐specific curves did not differ significantly by log-rank test (p=0.12), a finding that may reflect limited sample size despite clinically meaningful trends.

## Discussion

In our single-center Greek cohort, BCS with guideline-concordant radiotherapy yielded oncologic outcomes equivalent to mastectomy. This mirrors the 20- and 25-year follow-ups of NSABP B-06 and B-04 trials, which demonstrated no survival benefit for more extensive surgery [3]. Recent registry analyses, including a 30-year SEER study of over 500,000 women (HR: 0.87 for BCS vs. mastectomy) [10], propensity-matched NCDB cohorts showing a 10-15% relative mortality reduction with BCS [11], and Nedernalnd population studies have consistently favored breast conservation when radiotherapy follows standard protocols [12]. In our multivariable model, a nonsignificant trend toward higher risk with mastectomy (HR: 2.27, p=0.50) further supports that the extent of resection alone does not drive survival.

Our two surgical cohorts were not randomized and differed markedly at baseline. Patients undergoing mastectomy had larger tumors (57.5% vs. 26.6% in the 2-5 cm category and 15.0% vs. 0% >5 cm; p<0.001), more advanced stage (77.5% vs. 24.1% stage ≥2; p<0.001), and uniformly high grade (100% vs. 79.8% grade ≥2; p=0.006). They also showed greater lymphovascular invasion (50.0% vs. 26.6%; p=0.011), higher node‐positivity (50.0% vs. 11.4%; p<0.001), and different histology (ductal subtype 97.5% vs. 81.0%; p=0.013). Treatment patterns likewise diverged: mastectomy patients more often received NAC (35.0% vs. 15.2%; p=0.013), fewer completed adjuvant radiotherapy (25.0% vs. 100%; p<0.001), and were more likely to undergo full axillary dissection rather than sentinel‐node biopsy (85.0% vs. 19.0%; p<0.001). Additionally, molecular‐subtype distributions differed (p=0.037). These factors both drive the decision for mastectomy and independently worsen prognosis, introducing selection bias. Although our multivariable model adjusted for stage and subtype, residual confounding by treatment differences and unmeasured tumor or patient factors cannot be excluded. Acknowledging these imbalances underscores the need for cautious interpretation of unadjusted survival trends and highlights the value of prospective, randomized comparisons.

Pathological stage emerged as the dominant prognostic factor: higher stage conferred an eight-fold increased risk of death (HR: 8.32), far outweighing the effects of age, grade, or surgical type. This aligns with the Early Breast Cancer Trialists’ Collaborative Group meta-analysis of 42 trials, which showed that systemic tumor burden, captured by stage, accounts for the vast majority of breast cancer mortality, whereas variations in local treatment explain only a small fraction of outcome differences [2,4]. Our institutional protocols for staging and systemic therapy adhere to these data and international guidelines, ensuring treatment is calibrated to overall risk.

Intrinsic molecular subtype further modulates locoregional behavior and should inform shared decision-making. Although our subtype-specific survival curves did not reach statistical significance (p=0.12), their trends match larger datasets of basal-like (triple-negative) tumors recur earlier and more frequently, whereas luminal and HER2-enriched subtypes exhibit more durable control [13]. Voduc et al. reported a two-to-three-fold higher 10-year locoregional recurrence risk for basal-like disease even after adjustment for stage and treatment, and Freedman et al. documented a 10% five-year recurrence rate in modern triple-negative cohorts despite contemporary radiotherapy [13,14]. Conversely, trastuzumab has halved distant recurrence and mortality in HER2-positive disease, reflected by the plateau of our HER2-enriched survival curves [15]. At our center, surgical planning and adjuvant protocols follow these randomized trials and NCCN guidelines, ensuring evidence-based practice [5].

Landmark randomized trials reinforce these conclusions. In EORTC 10801, 701 women with tumors ≤2 cm were randomized to quadrantectomy plus whole-breast radiotherapy versus modified radical mastectomy; at 10 years, overall survival was virtually identical (66.1% vs. 66.9%), with no significant difference in locoregional recurrence [16]. CALGB 9343 randomized women aged ≥70 with hormone-receptor-positive tumors ≤2 cm to lumpectomy plus tamoxifen with or without radiotherapy; at 10 years, overall survival remained equivalent (67% vs. 66%), despite a modest increase in local recurrence in the no-radiotherapy arm [17]. The ACOSOG Z0011 trial showed that, among sentinel-node-positive patients undergoing lumpectomy and whole-breast radiation, omission of completion axillary dissection did not compromise 10-year overall survival (83.6% vs. 82.2%), highlighting safe de-escalation of surgical extent when combined with appropriate radiotherapy [18].

The use of NAC increasingly drives surgical decision-making, not only for breast but also for the axilla. Pivotal studies ACOSOG Z1071 and SENTINA demonstrated that marking the biopsied positive node and performing targeted axillary dissection (TAD) plus sentinel‐node biopsy yields a false-negative rate below 5% in patients who convert from cN+ to ycN0 after NAC [19,20]. The preliminary data show striking heterogeneity in axillary-staging practices among participating countries. After only 20 months of recruitment, more than half of the target cohort (3,000 patients) has been enrolled. While TAD is widely adopted, both axillary lymph node dissection (ALND) and SLNB remain common. The AXSANA study will determine whether de-escalation of axillary surgery is safe for patients who convert from clinical node-positive (cN+) to ycN0 status following NAC [21]. Meanwhile, the Alliance A011202 trial is evaluating whether TAD can safely replace full ALND in this setting, potentially sparing many women the morbidity associated with complete dissection. Notably, omission of ALND in patients with residual nodal disease after NAC is on the rise, especially in community centers and among those with a lower burden of residual disease. To date, no impact on OS has been observed. Integrating TAD into our protocols will better align the extent of surgery with each patient’s response to systemic therapy, further personalizing care [22].

More recent phase III studies continue to refine management. The SOUND trial randomized 1,405 women with tumors ≤2 cm and negative axillary ultrasound to sentinel-node biopsy versus no axillary surgery; five-year distant disease-free survival was virtually identical (97.7% vs. 98.0%), with no difference in OS [23]. KEYNOTE-522 enrolled 1,174 patients with stage II-III triple-negative breast cancer, randomizing them to pembrolizumab plus neoadjuvant chemotherapy (and adjuvant pembrolizumab) versus chemotherapy alone; at 75 months, five-year OS was significantly higher in the pembrolizumab arm (86.6% vs. 81.7%; p=0.002) [24]. The monarchE trial involving 5,637 high-risk ER+/HER2- patients showed that two years of adjuvant abemaciclib plus endocrine therapy improved invasive disease-free survival (IDFS) (HR: 0.68; 95% CI: 0.60-0.77) and distant relapse-free survival, yielding a 7.6% absolute benefit at five years [25]. Our center has incorporated these advances, including immunotherapy for triple-negative disease and CDK4/6 inhibition for high-risk ER+ patients, into our multidisciplinary protocols per ASCO and ESMO guidelines.

Targeted and perioperative innovations further enhance our approach. For HER2-positive metastatic disease, we adopted fam-trastuzumab deruxtecan (Enhertu), whose superiority over T-DM1 was established in DESTINY-Breast 03 [progression-free survival (PFS) HR: 0.28; OS HR: 0.55] [26,27]. In the adjuvant setting, germline BRCA1/2 carriers now receive one year of olaparib per OlympiA (IDFS HR: 0.58; OS HR: 0.68) [28]. Surgically, we routinely employ oncoplastic volume-displacement and replacement techniques, allowing wider resections with low positive-margin rates, and use high-resolution MRI and image-guided localization preoperatively [29]. Postoperatively, our Enhanced Recovery After Surgery (ERAS) pathways (multimodal analgesia, early mobilization, structured wound care) have safely shortened length of stay and reduced opioid use [30]. Embedding these therapies and modern surgical protocols ensures our patients benefit from the highest standards of care.

Clinical implications in the Greek setting are clear: BCS should remain the default for early-stage tumors when anatomical and patient-preference criteria permit. Mastectomy retains its role for multifocal or extensive disease, prior chest irradiation, or high-penetrance germline mutations, but not as a surrogate for oncologic safety. Basal-like cancers warrant margin-negative surgery combined with optimized systemic therapy and vigilant surveillance.

Strengths and limitations

The strengths of the study include the adoption of centralized pathology review, contemporary molecular subtyping, and complete follow-up. However, it has certain limitations as well, such as its retrospective design, limited event numbers for subtype-specific analyses, and absence of patient-reported outcomes (cosmesis, quality of life). Future multi-institutional Greek registries incorporating genomic platforms and patient-centered metrics will enable propensity-matched analyses to further refine locoregional recommendations.

Future directions

Prospective, subtype-specific trials are urgently needed to evaluate de-escalation strategies such as limited axillary dissection in node-negative, luminal tumors, and escalation approaches, including regional nodal radiotherapy or immunotherapy for basal-like cancers. Simultaneously, incorporation of real-world evidence related to treatment costs, cosmetic outcomes, and patient-reported satisfaction will be crucial for developing a biology-driven surgical paradigm that preserves oncologic efficacy while enhancing quality of life.

## Conclusions

The findings of our single-center analysis confirm that BCS with standard-of-care adjuvant radiotherapy delivers long-term survival outcomes at least equivalent to mastectomy. We demonstrate that pathological stage and intrinsic molecular subtype, rather than the choice of lumpectomy versus mastectomy, are the principal determinants of prognosis, underscoring the primacy of early detection and optimized systemic therapy. By integrating modern targeted agents and advanced oncoplastic techniques within a structured ERAS pathway, our multidisciplinary protocol embodies the highest standards of evidence-based, patient-centered care. Going forward, ongoing and future subtype-driven de-escalation and escalation trials will further refine our ability to tailor surgical and systemic interventions. Collectively, these data support a personalized, biology-driven paradigm in breast cancer management that maximizes oncologic safety, preserves quality of life, and continually adapts to emerging therapeutic innovations.
